# Evaluating new types of tourniquets by the Israeli Naval special warfare unit

**DOI:** 10.1186/2054-314X-1-1

**Published:** 2015-01-27

**Authors:** Eitan Heldenberg, Shahar Aharony, Tamir Wolf, Tali Vishne

**Affiliations:** 1 Department of Vascular Surgery, Assaf Harofeh Medical Center, affiliated to the Sackler faculty of medicine, Tel Aviv University, Tel Aviv, Israel; 2 Israeli Naval Special Warfare Unit, Israeli Navy, Isreal Defence Forces, Kragujevac, Israel; 3 Department of Psychiatry, Maayanei Hayeshouha Medical Center, Bnai Berak, Israel

**Keywords:** External bleeding, Tactical combat casualty care, Combat application tourniquet, Special operations force tactical tourniquet

## Abstract

**Background:**

Extremity injuries, which accounts for 20% of all battlefield injuries, result in 7-9% of deaths during military activity. Silicone tourniquets were used, by the Israeli Defense Force (IDF) soldiers, for upper extremity and calf injuries, while thigh injuries were treated by an improvised "Russian" tourniquet (IRT). This is the first study, performed in the IDF, comparing the IRT with Combat Application Tourniquets (CAT) and Special Operations Force Tactical Tourniquets (SOFTT). 23 operators from the Israeli Naval Unit (Shayetet 13) were divided into two groups according to their medical training (11 operators trained as first-responders; 12 operators as medics). Repetitive applications of the three tourniquets over the thigh and upper arm, and self-application of the CAT and SOFTT over the dominant extremity were performed using dry and wet tourniquets (828 individual placements) with efficacy recorded. Cessation of distal arterial flow (palpation; Doppler ultrasound) confirmed success, while failure was considered in the advent of arterial flow or tourniquet instability. Satisfaction questionnaires were filled by the operators.

**Results:**

CAT and SOFTT were found to be superior to the IRT, in occluding arterial blood flow to the extremities (22%, 23% and 38%, respectively, failure rate). The application was quicker for the CAT and SOFTT as compared to the IRT (18, 26, 52 seconds, respectively). Wet tourniquets neither prolonged application nor did they increase failure rates. Similarly, medics didn't have any advantage over non-medic operators. No findings indicated superiority of CAT and SOFTT over one another, despite operators’ preference of CAT.

**Conclusions:**

CAT and SOFTT offer an effective alternative to the IRT in stopping blood flow to extremities. No difference was observed between medics and non-medic operators. Thus, the CAT was elected as the preferred tourniquet by our unit and it is being used by all the operators.

## Background

The extremities are the primary injury site in military trauma, at times posing an immediate threat to the soldier's life [1]. During the Vietnam war up to 74% of penetrating injuries involved the extremities, with a related mortality rate as high as 10% [2], The Israeli experience from the Yom-Kippur war (1973) indicates that extremity injuries were approximately 60% of all injuries [3]. Recently gathered data from Operation Iraqi Freedom (OIF) and Operation Enduring Freedom (OEF) (in Afghanistan) as well as data gathered in Israel throughout the second Palestinian uprising (Intifada) [4, 5] indicates that 90% of military casualties are secondary to penetrating injuries [5]. Of those, 50-70% suffer extremity injuries, with mortality rates of up to 9%, mainly due to improper treatment of external hemorrhage [5, 6]. Uncontrolled external hemorrhage of the extremities, currently referred as compressible bleeding, is the primary cause of preventable deaths in the field.

The Israeli pre-hospital care givers have adopted a very liberal approach to tourniquet usage is, both in military as well as in civilian settings. The civilian approach, most probably influenced by the military one, sees the tourniquet as an essential tool for primary treatment of compressible bleeding. On the other hand there are primordial fears of further tissue damage (i.e., ischemic, neurological and following removal – reperfusion injury) that will eventually lead to amputation [7, 8].

The military practice encourages tourniquet usage in the battlefield. The principals presented as Tactical Combat Casualty Care (TCCC) unite tactical and medical considerations in tailoring causality treatment to the surrounding environment and threats [7]. In TCCC, tourniquet usage is considered a first-line treatment for controlling an ongoing extremities compressible bleeding. Thus, although pressure applied directly to the injury site may be preferable, inability to do so due to tactical considerations leads to tourniquet application as an early phase of treatment. The common indications for the tourniquet usage are care under fire, dark environment, as well as "medical" considerations including failure of previously applied direct pressure, traumatic amputation, multiple bleeding sites along one limb, and a foreign body piercing the limb thus preventing application of direct pressure [7, 8].

During their basic training the IDF soldiers undergo “First responder” course during which they are being qualified to apply tourniquet. The IDF Medical Corps used two forms of tourniquets – the silicone strap and the “improvised Russian tourniquet” (IRT), which was used for thigh injuries. The elastic silicone strap is used for upper extremity and calf injuries. Successful application is verified by cessation of bleeding and lack of pulse distal to the injured site. The exact mechanism responsible for the bleeding cessation is not clearly defined, however it is most probably secondary to an increased compartmental pressure which squeezes the vascular system. This seems to be the explanation to the suggested application pressure of 50-150 mmHg above the systolic blood pressure [9, 10]. A field operator should be taught the different types of bleeding, the arterial – the pulsatile one and the venous – the continuous one, in order to overcome a common pitfall, which is the caseation of venous bleeding, for which lower external pressure is needed, as compared to arterial one. A patient in shock, with a temporary caseation of arterial bleeding due to a thrombosed artery, might re-bleed due to thrombus dislodgement when the blood pressure will increase if not enough external pressure will be applied.

As a results of the above suggested mechanism, the silicone tourniquet is not effective for thigh arterial injuries, due to the larger muscle mass, which serves as a barrier between the tourniquet and the thigh arteries.

In order to handle thigh injuries the IDF has adopted an 18^th^ century improvisation, known in Israel as the IRT, and in the US military as the “Spanish Windlass” [9]. It is composed of a wooden rod and two triangular bandages, used to exert pressure on the limb proximal to injury site. There are several major drawbacks to this device: its storage and carriage are not users friendly, its application is quite clumsy (even more so when self-application is attempted) and since it's an improvisation, there is no consensus on how it should be applied.

The American experience in OIF and OEF was the major initiative for a breakthrough in tourniquet technology. The need for an effective self-applied tourniquet, yielded an evaluation of *nine* newly developed devices that met the Request for Information (RFI) criteria set by the US Army Institute of Surgical Research. Three tourniquets - the Combat Application Tourniquet (CAT; *Phil Durango LLC*), the Special Operations Force Tactical Tourniquet (SOFTT; *Tactical Medical Solutions LLC*) and the Emergency Military Tourniquet (EMT; *Delfi Medical Innovations, Inc*) - were found to be the most effective. Subsequently, the US military branches began using the CAT and SOFTT tourniquets as their preferred devices [11].

The Israeli Naval Special Warfare Unit (“Shayetet 13”; henceforth: INSWU) is a very dynamic force, operating in various combat settings. All INSWU combat team members undergo advanced first-responder training, given that, they would usually provide the first assistance to the injured teammate, due to tactical reasons.

The study's objectives were to compare the efficacy of the CAT and the SOFTT to the IRT and to define the preferred tourniquet for all the unit's operators (medics and non-medics). Our hypothesis was that the CAT and SOFTT will be the preferred types of tourniquet since they are much easier to apply.

## Materials and methods

### Study design

The study, which was conducted at the INSWU base, was a prospective observational study. IRB approval was obtained from the Israeli Medical Corps Review Board. The study participants were instructed by the unit's physicians about the scope of the study, the procedures to be done and possible complications. The participants, 23 healthy males, combat teams members, aged 19-23, gave their written informed consent to participate in the study.

The combat team members were divided into two subgroups: 11 non-medic members, with advanced first-responder training, and 12 medics.

Three tourniquet types were assessed in this study: the IRT, the CAT and the SOFTT. The silicone strap was not assessed in this study since it does not qualify for the treatment of thigh injuries. All the participants were instructed, by the unit's physicians, regarding the different types of tourniquets to be tested, and this was followed by self-application session.

Each assessment was performed with both dry and wet tourniquets, in order to simulate as much as possible the different scenarios, in which the tourniquet might be applied by the INSWU members.

The participants’ baseline data, including height, weight and limb circumference (mid-thigh and mid-upper arm), blood pressure and heart rate were recorded, prior to the tourniquet application.

Each participant applied all three types of tourniquets on one of his peers. This was followed by self-application of the CAT and the SOFTT systems on the dominant arm. The participants were instructed to continue tightening the tourniquets as per the instructions for use (IFU). The unit's physicians did not interfere with the tourniquet application process.

The following parameters were assessed by the participants: application technique simplicity, self-application comfort, storage comfort, overall device simplicity, pain during application. The following parameters were assessed, by the assigned physician, in order to verify correct application: palpable pulse and audible Doppler signals (Medlink, Bidirectional Doppler, DP 2000, France) over the Radial and Ulnar arteries (upper limb applications) and over the Dorsalis Pedis and Posterior Tibial arteries (lower limb applications). Following correct application, the tourniquet was slowly released to confirm re-establishment of Doppler signal (ensuring that the lack of signal was indeed secondary to the tourniquet and not to incorrect probe-misplacement). Application duration was assessed for each type of tourniquet tested.

Failure was recorded in case of a palpable pulse, audible Doppler signal, or mechanical failure (tourniquet instability). All of these following the participant declaration that he has completed his task.

Finally, all the participants filled out questionnaires in which subjective parameters including satisfaction, user-friendliness, storage, simplicity, and pain were assessed.

### Data analysis

Baseline subject characteristics and questionnaire analysis were performed using Students’ *t*-test. A total of 828 tourniquet applications were performed throughout the study. Time of tourniquet application was analyzed similarly, using t-test, following logarithmic correction in order to normalize the results to the Gaussian curve. Percentage of failure was assessed using the Chi-square test. Significance was set at *p* < 0.05. All results are given as a mean ± SD.

## Results

**Figure 1:**
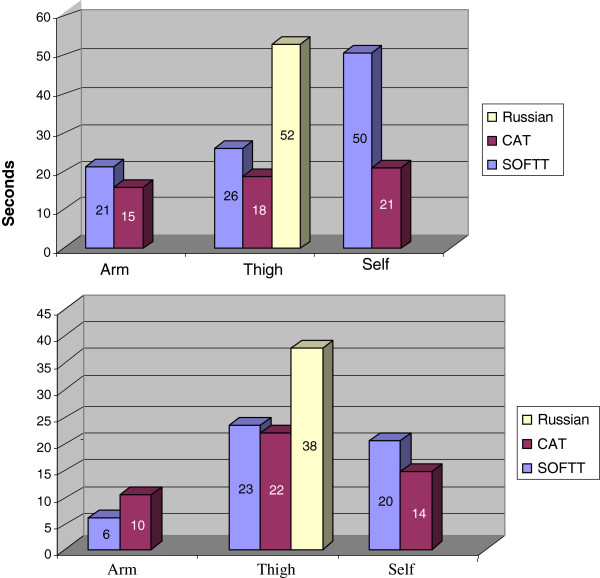
**Average time (sec.) of tourniquet's application.** Failure rates (%) of tourniquet application.

The CAT had the highest assessment score by the operators, followed by the SOFTT and IRT (4.6±0.6, 4.0±1.0, 2.1±1.0, respectively) (Table 1). Both arm as well as the self-application, were faster for CAT as compared to SOFTT (13 ± 4 sec and 21 ± 8 sec versus 18 ± 7 sec and 54 ± 69 sec, respectively). CAT and SOFTT thigh applications were much quicker (19 ± 7 sec and 24 ± 7 sec, respectively) as compared to the IRT, which on average took at least twice as long to place (53 ± 23 sec) (Figure 1). The IRT thigh application failure rate was 38%, as compared to 22% and 23% for the CAT and SOFTT, respectively. SOFTT arm application failure rate was lower than the CAT application failure rate (6% and 10%, *p* = 0.266). CAT application failure rate was lower when self-application was used (SOFTT 20%, CAT 14%, *p* = 0.5) (Figure 1).

**Table 1:** **Operators tourniquet assessment**

	SOFTT	CAT	IRT
Application technique simplicity	4.0 ± 0.8	5.0 ± 0.2	2.1 ± 0.8
Self-application comfort	3.1 ± 1.3	4.5 ± 0.6	NA
Storage comfort	4.4 ± 0.8	4.6 ± 0.6	2.3 ± 1.1
Overall device simplicity	4.0 ± 0.8	4.7 ± 0.5	2.6 ± 1.3
No pain during application	4.1 ± 0.7	4.1 ± 0.6	1.3 ± 0.5
Summarized score	4.0 ± 1.0	4.6 ± 0.6	2.1 ± 1.0

**Figure 2:**
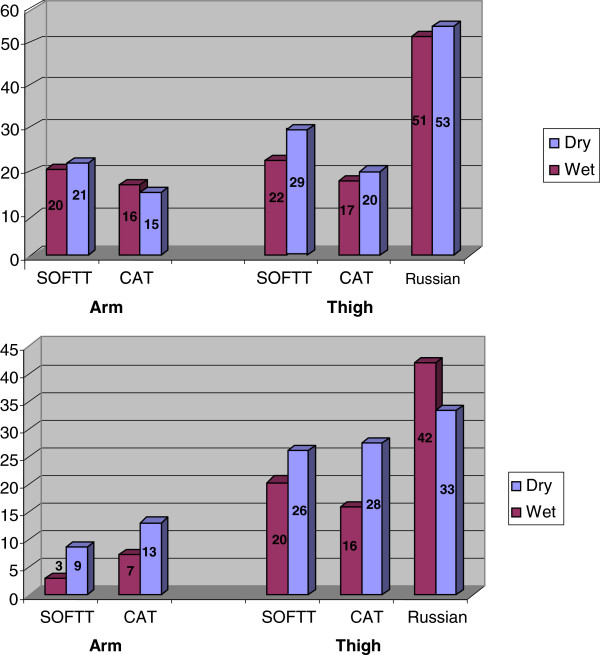
**Average time (sec.) of tourniquet application based on dampness.** Failure Rate (%) of tourniquet application based on dampness.

**Figure 3:**
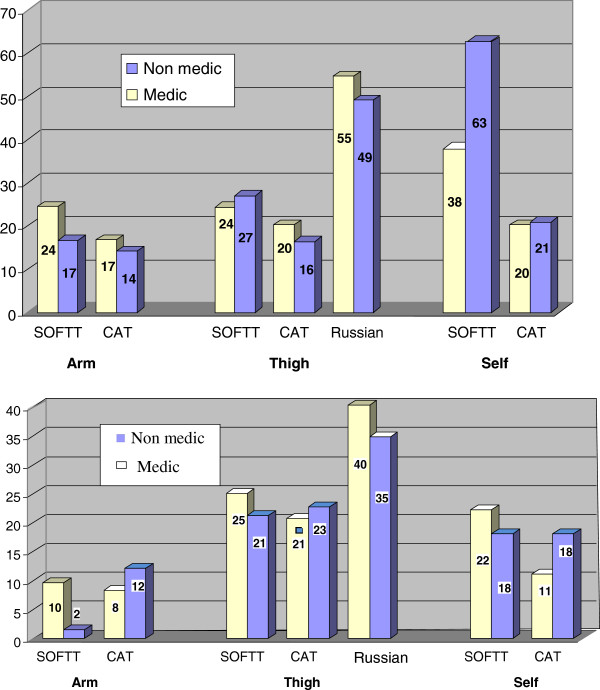
**Average time (sec.) of tourniquet application based on operator.** Failure Rate (%) of tourniquet application based on operator.

No evidence demonstrating that wet tourniquets either prolonged application time or increased tourniquet application failure rate, at all anatomical sites, was found (Figure 2). Medics had no advantage as compared to the non-medic operators regarding tourniquet's application (Figure 3). Generally, non-medic operators placed the tourniquets faster, though medics were quicker in self-applying the SOFTT (37 ± 58 sec as opposed to 55 ± 69 sec, *p* = 0.236). Operator failure rates while applying arm CAT were higher as compared with the SOFTT application (12% versus 2%, *p* < 0.04). Failure rates of the improvised tourniquet application (35%) were higher as compared with both the CAT and SOFTT (23 and 21%, respectively), though without statistical significance. No difference was found in self-application failure rate (18%),of the latter two tourniquets. Medic failure rates of CAT and SOFTT arm application did not differ (8% and 10%, respectively, *p* = 1). Thigh CAT application was more effective than that of the IRT (21% and 40% failure, respectively, *p* = 0.019). Medics’ CAT self-application was more effective than SOFTT (11% versus 22% failure, respectively) but without statistical significance.

The participant's assessed of the tourniquets’ manipulation and storage parameters in a scale of 1-5 (1- the lowest score and 5 – the highest one). The CAT was assessed as the preferred device (a score of 4.6 ± 0.6), followed by the SOFTT (4.0 ± 1.0) and the IRT (2.1 ± 1.0) (p < 0.0001).

## Discussion

Extremity injuries comprise 20% of all battlefield injuries which may lead to exsanguinating bleeding in 7-9% of the casualties [3].The medical response to orthopedic and mainly vascular injuries, in the ^.^field, is obviously inferior to the one being given once the patient arrives to the hospital. In order to minimize mortality due to such injuries, external extremity bleeding control, using tourniquets is sometimes mandated.

The Low Intensity Conflict during the recent years, which has become the modern battlefield (the Intifada, OEF & OIF), confronts military forces with semi-organized guerrilla forces, mostly in urban setting [5, 9]. Such settings, where the fighting arena has shifted to narrow street alleys, are characterized, by an abundant of personal weapons, grenades and mines as well as improvised explosive devices (IED). These settings, coupled with technological developments of personal armor systems that protect the torso, and advances in head and neck protection gear are a major cause for the increased percentage of extremity injuries among soldiers [12–14].

As those penetrating extremity injuries became the major concern of the medical caregivers, a lot of attention was focused on modalities to stop the ongoing bleeding. The use of tourniquet is the most important maneuver saving most of the casualties’ lives in the battlefield.

Throughout the past two decades, the IDF has adopted a permissive approach, enabling a liberal tourniquet use. Soldiers are trained, on a regular basis, how to correctly apply the tourniquet. Combat team members, as well as medics and physicians, carry tourniquets in their pockets, thus enabling them to immediately treat on going external bleeding. The medics used to carry an improvised tourniquet, which was superior to the, previously standard-issued, silicone one in stopping external thigh bleeding. For many years no significant technological development had been made enabling utilization of something better than the IRT. Only during the beginning of the current century the experience gathered throughout OIF and OEF by the US military was a major catalyst for such a development, which promoted the development of several tourniquets superior to the improvised ones in previous use [14–16].

Lakstein reported a 72% success rate, by IDF, with the IRT in relieving bleeding. Though no differences were observed between physicians, medics, and soldiers, the authors felt that trained medical personnel would probably be more successful, and lack of statistical significance between caregivers is due perhaps to the small sample size (23 subjects) [5].

King et al., compared five various tourniquet systems including the pneumatic EMT, a latex surgical tubing (ST) tourniquet, and an improvisation similar to the one utilized in the IDF. They concluded that the ST, despite its shortcomings, should be distributed freely to all soldiers, and the EMT tourniquet should be issued only to medics trained in its use [17, 18].

As was mentioned earlier, the American involvement in OIF and OEF was the major catalyst for the development of new types of tourniquets, mainly the CAT and the SOFTT.

Walters et al., demonstrated a success rate of 100% in reducing thigh arterial blood flow, comparing CAT and SOFTT applications in the laboratory settings [11].

Lairet et al demonstrated in a large study of 1003 casualties in Afghanistan, that tourniquet application had the lowest percentage of missed or incorrectly done intervention in the battlefield [19]. Beekley et al demonstrated in OIF casualties that prehospital tourniquet use was associated with improved hemorrhage control, particularly for the worse injured (ISS > 15) subset of patients. They concluded that 57% of the deaths might have been prevented by earlier tourniquet use [20].

The INSWU reaches its targets through the sea, either above or below the water level. As such, there is a major issue regarding the manipulation of wet tourniquets over wet body regions. The applications were performed by combat team members, medics and non-medics, at various anatomical regions, using both dry and wet tourniquets. These simulate the fields’ conditions, where tourniquets are applied over a bloody and slippery extremity surface. In the study described herein, tourniquets were applied in a controlled environment, the INSWU base. The controlled environment, in which this study was performed, is its major advantage as well as its major limitation although we have tried to simulate the true scenarios our combat teams are dealing with.

The current study demonstrated a statistically significant superiority of the CAT and SOFTT tourniquets as compared to the IRT in all the assessed parameters.

Lakstein found superiority of the IRT over the silicone one in controlling thigh bleeding, with 72% and 66% success rates, respectively [5]. The current study demonstrated 62%, 78%, and 77% success rates using the IRT, CAT and SOFTT tourniquets, respectively. How can Lakstein’s, relatively high, success rate be explained? The following factors might resolve the dilemma: A high percentage of tourniquet applications in Lakstein's study were not performed in compliance with IDF Medical Corps indications (47%); no active bleeding was reported in 75% of the described injuries prior to tourniquet application. Lakstein describes real world results of injured hypovolemic patients, with lower blood pressure measurements, to which the tourniquets were applied (i.e., less pressure is required in order to stop the bleeding [5]. On the other hand these differences may be secondary to sampling error, in a small study group, and not reflect clinically significant difference.

The current study demonstrated lower success rates while using the same types of tourniquets. Since our participants followed the IFU by the manufacturers'. It seems that our results reflects better the true incidence of failure rate.

In order to minimize bias, no interference was made by the ISNWU physicians while the participants applied the tourniquets. Failures stemmed from technical errors or insufficient pressure exerted. We elected, as in previous studies, to assess the tourniquet application, using Doppler signals. The disappearance of the Doppler signals sound indicated a correct tourniquet application [21, 22]. The Doppler was used, in those studies, as a direct en-route feedback mechanism throughout the application process. In order to simulate the battlefield area as much as possible, Doppler assessment was conducted in our study, only once the participants indicated they had finished the tourniquet application.

Our relatively lower success rate compared to Walter’s group can be explained by the fact that our study was conducted on non-injured patients. Active bleeding, which usually serves as feedback parameter for the quality of tourniquet application, could not be used to assess the usefulness of the tourniquet application. The current study involved special operations force operators, who tend to have a higher muscle mass, requiring more pressure to compress the thigh arteries. These issues were specially addressed by the unit's physicians and the operators (medics and non-medics) were instructed about proper tourniquet application. It worth mentioning that according to new lessons learned from OIF & OEF, in some cases of very athletic muscular young combat team members, more than one tourniquet may be needed in order to control the bleeding.

Our results did not find higher failure rates in wet Vs. dry applications, in contrary to one assumption that wet application would have a higher failure rates. These results might be related to the awareness of the unit's physicians and instructed lessons, regarding wet tourniquet application, given to the unit's operators.

Previous studies indicated a trend in which medically trained personnel had a distinct advantage over those without formal training, leading to higher application success rates [20–23]. No difference was found, between the medics and non-medic in our groups, as regard to either application time, or in their failure rates. This finding is of extreme importance, as one of the goals of this study was to define the best tourniquet for use, by all combat team members, not only by trained medical personnel. It either reflects the easiness and simplicity of the new type of tourniquet (CAT & SOFTT) or it reflects the good training of the non-medics in the INSWU in regards to tourniquet application.

As expected it was found that for all anatomical locations, CAT and SOFTT applications were much faster than IRT application. This was especially true, and may be of clinical significance, regarding application on the thigh (18 sec and 26 sec as opposed to 52 sec). The importance of quick action in the field, especially under fire, cannot be over-emphasized.

The combat team members of the INSWU were the study population for which this study was originally designed. Hence, questionnaires assessing their personal preferences regarding application technique, future storage options, pain experienced following the tourniquet application, and overall user-friendliness were handed out. Both the CAT and SOFTT received significantly higher scores than the IRT in all indices. CAT was considered by our warriors a more user-friendly device than the SOFTT.

## Conclusion

The CAT and the SOFTT, were more effective by both medically trained personnel and other members as compared to the older types of tourniquet. Water had no impact on failure rate – the same failure rate was noticed for either dry or wet applications. Despite the participants’ preference of the CAT, no objective finding indicated a significant superiority over the SOFTT. A larger study should be held in the future, in order to examine if there is any superiority of one of those two tourniquets over the other.

## Abbreviations

CAT: Combat application tourniquet
IDF: Israel defense force
INSWU: Israeli Naval special warfare unit
OEF: Operation enduring freedom
OIF: Operation Iraqi freedom
SOFTT: Special operations force tactical tourniquet
TCCC: Tactical combat causality care.
